# Detection and Quantification of Total and Pathogenic *Vibrio parahaemolyticus* in *Anadara subcrenata* in the Zhoushan Archipelago

**DOI:** 10.1155/2019/5481935

**Published:** 2019-11-25

**Authors:** Bing Wu, Hongxia Gong, Hui Zhang, Jiabei Chen, Hongling Wang

**Affiliations:** ^1^Key Laboratory of Health Risk Factors for Seafood of Zhejiang Province, Zhoushan, Zhejiang, China; ^2^Zhoushan Municipal Center for Disease Control and Prevention, Zhoushan, Zhejiang, China

## Abstract

This study aimed to investigate the prevalence of total and pathogenic *Vibrio parahaemolyticus* in *Anadara subcrenata* sampled from aquafarms and retail markets in the Zhoushan islands during June 2013 to March 2015, using the most probable number-polymerase chain reaction (MPN-PCR) method. Total *V. parahaemolyticus* was detected in 265 (83.86%) samples with the density 0.3 to 2400 MPN/g. In total, 30.70% and 17.41% of the samples exceeded 100 MPN/g and 1,000 MPN/g, respectively. Both highest positive rate (98.99%) and highest prevalence (median = 210.0 MPN/g) were recorded in summer. Samples from aquafarms had a higher positive rate and median than those from retail markets. Pathogenic *V. parahaemolyticus* was detected both in aquafarms and retail markets in all seasons but not in winter. Among the 265 *tlh*-positive samples, 20 (7.55%) of the samples harbored *tdh*, and 5 (1.89%) of the samples harbored both *tdh* and *trh*. These results indicate that the Zhoushan archipelago is severely contaminated with *V. parahaemolyticus* in *Anadara subcrenata*; these results are applicable in risk assessment and to control the risk of food-borne disease caused by *V. parahaemolyticus*.

## 1. Introduction

*Vibrio parahaemolyticus* is a marine microorganism, recognized as a primary pathogen of seafood-borne illnesses in North America, Europe, and Asia [1–4]. In recent years, *V. parahaemolyticus* has also become the primary pathogen causing food-borne gastroenteritis in coastal cities of China [4]. The Zhoushan archipelago, East China Sea, is famous for its abundant fishery resources and sceneries for tourists. Seafood, especially blood clam (*Anadara subcrenata*), is the principal food among local residents. However, *V. parahaemolyticus* has been reported to be commonly present in the culture environment used to grow shellfish, including blood clam [2]. Individuals are faced with a high risk of bacterial infection via consumption of raw or uncooked shellfish, especially blood clam, which is considered the primary cause of *V. parahaemolyticus* infection [1].

In the United States, consumption of raw or undercooked shellfish results in approximately 35,000 domestically acquired food-borne *V. parahaemolyticus* infections annually [5]. Many infections are epidemiologically associated with the consumption of shellfish contaminated with pathogenic strains [6]. Therefore, controlling *V. parahaemolyticus* contamination in seafood can effectively prevent food-borne disease, and it is important to determine the levels of contamination of *V. parahaemolyticus* both in aquafarms and in retail markets [7].

The pathogenesis of *V. parahaemolyticus* infections has been studied extensively, and the thermostable direct hemolysin (TDH) encoded by *tdh* and TDH-related hemolysin (TRH) encoded by *trh* are considered pathogenic genes which are present mostly in clinical strains [8]. Recognized as primary virulent factors of *V. parahaemolyticus*, either or both toxins encoded by *trh* and *tdh* can cause illness [9]. Several methods have been described to determine levels of contamination of *V. parahaemolyticus*; however, the results have been inconsistent in accordance with the analytical method.

Thus, this study aimed to detect and quantify the total and pathogenic *V. parahaemolyticus* in blood clam from aquafarms and retail markets in the Zhoushan islands, using the most probable number-polymerase chain reaction (MPN-PCR) method [10], analyzed the contamination levels of *V. parahaemolyticus*, and provide evidence for risk control of food-borne disease.

## 2. Materials and Methods

### 2.1. Samples

From June 2013 to March 2015, 316 *Anadara subcrenata* samples were collected between 10th to 19th every month from aquafarms and retail markets in Zhoushan. Each month, 5–10 samples were purchased from the primary 2 retail markets (Donghe markets in the Putuo district and Qiandao market in the Dinghai district) and 3 seafood restaurants (Xiangnan Hotel and Jiaotong Hotel in the Dinghai district and Yihao Yugang in the Putuo district), and 5–10 samples were collected from two aquafarms (Dongshao aqua farm and Daidong aqua farm, 30.28N, 122.22E) in the Daishan island (Table 1). Each sample consisted of 10–12 individual clams, but dual to the size difference, sample weight ranges from 100 g to 300 g. All samples were placed in sterile, labeled, sealed plastic bags on ice and were immediately analyzed on the day of sampling.

### 2.2. MPN-PCR Method

The MPN-PCR method was used to estimate *V. parahaemolyticus* in 3 density levels in the samples [11]. Twenty-five Gram samples were fully cut into pieces with aseptic scissors in homogeneous cup after separated from shells and combined with 225 mL of alkaline peptone water (APW) containing 3% NaCl (Huankai, Guangzhou, China) to generate a 1 : 10 dilution. Three serial 10-fold dilutions were applied (10^−1^ to 10^−3^), and all dilutions were incubated at 37°C for 16–18 h. In total, 1.5 vmL of the MPN culture was centrifuged and resuspended twice. DNA was extracted by resuspending samples in 200 *μ*L of DNA extraction buffer (Zhuocheng, Shanghai, China) and heated in 100°C for 10 min. The suspension was centrifuged at 3,000 ×g for 1 min, and the supernatant was used as the template DNA. *V. parahaemolyticus tlh, tdh*, and *trh* were detected via Roche LightCycler® 480II real-time PCR system. The primer pairs and probes used for detection are enlisted in Table 2, and the reactions were performed as described by Ward et al. [12].

The PCR cycling conditions were as follows: denaturationat 95°C for 120 s, followed by amplification for 45 cycles, each comprising denaturation at 95°C for 20 s, annealing at 56°C for 20 s, and extension at 72°C for 30 s. The fluorescence signal was measured and recorded at the end of the annealing step of each cycle.

### 2.3. Statistical Analysis

The MPN value was normalized before analysis. The MPN value was normalized as follows: the MPN value under 0.30 MPN/g was estimated to be 0.15 MPN/g; over 110 MPN/g, 240 MPN/g; and over 1100 MPN/g, 2400 MPN/g [10]. In accordance with the month of sampling, all samples were classified into four seasons: spring (March to May), summer (June to August), autumn (September to November), and winter (December to February). Samples were also determined on the basis of two sampling resources: aquafarm and retail market. The results were analyzed using Pearson's chi-squared test for the prevalence of total and pathogenic *V. parahaemolyticus*. To compare differences among sampling seasons and methods, considering nonnormal distribution of the resulting data, two non-parametric tests (the Levene's robust test and Kruskal–Wallis nonparameter test) were performed for analysis. The correlation coefficient between total and pathogenic *V. parahaemolyticus* contamination levels was determined via Pearson's correlation (Spearman's correlation) analysis. A *P* value of 0.05 was considered to determine statistical significance. Statistical analysis was performed using Microsoft Excel add-in tools and the SPSS 20.0 software.

## 3. Results

The incidence and contamination levels of total *V. parahaemolyticus* in *Anadara subcrenata* are enlisted in Tables 3 and 4. Among the 316 samples, 265 (83.86%) were tested positive for *tlh*, thereby being identified as *V. parahaemolyticus*-positive. Levels of total *V. parahaemolyticus* vary from <0.3 to 2400.0 MPN/g with median 42.0 MPN/g. The samples of *V. parahaemolyticus* at each level (<10, 10-1 × 10^2^, 1 × 10^2^-1 × 10^3^, and >1 × 10^3^ MPN/g) were 71, 97, 42, and 55. In total, 30.70% and 17.41% of samples exceeded 100 MPN/g and 1,000 MPN/g, respectively.

Table 3 shows the median contamination levels of *V. parahaemolyticus* in *Anadara subcrenata* in all seasons. The positive rates among the four seasons were significantly different (*χ*^2^ = 38.371, *P* < 0.01). Both the highest positive rate (98.99%) and the highest contamination level (median = 210.0 MPN/g) were observed in summer, while the lowest positive rate and contamination levels were observed in winter. In accordance with nonparametric tests, contamination levels of total *V. parahaemolyticus* were significantly different (*χ*^2^ = 122.797, *P* < 0.001) among different seasons.

As shown in Table 4, the positive rate of *V. parahaemolyticus* in the samples from retail markets was 73.64%, which was lower than that of aquafarms (92.85%). The MPN/g estimate of *V. parahaemolyticus* in the samples from retail markets with the median 24 MPN/g was lower than that in aquafarms (53 MPN/g). For samples that exceeded 100 MPN/g and 1000 MPN/g, those fromaqua farms were 17.26% and 23.21%, greater than those in retail markets, respectively. The level of contamination was significantly different between aqua farms and retail markets total *V. parahaemolyticus* (*P* < 0.01).

Pathogenic *V. parahaemolyticus* strains were determined on the basis of the presence of one or both of the *tdh* and *trh* genes. Among the 265 *tlh*-positive samples, 7.55% (20 of 265) samples harbored *tdh*, and 1.89% (5 of 265) samples harbored both *tdh* and *trh*. The total positive rate of pathogenic *V. parahaemolyticus* was 9.43%. The contamination rate of pathogenic *V. parahaemolyticus* with the median 6.4 MPN/g ranged from <0.3 to 240 MPN/g.

Differently from total *V. parahaemolyticus* density, the highest density of pathogenic *V. parahaemolyticus* was generally detected in samples obtained during spring (Table 5). None of the samples obtained during winter tested positive. The positive rate of pathogenic *V. parahaemolyticus* differed significantly among different seasons (*P* < 0.05).

Apart from total *V. parahaemolyticus*, both positive rate and density of pathogenic *V. parahaemolyticus* in samples obtained from retail markets were greater than those obtained from aquafarms, being generally lesser than 10 MPN/g for both samples, which are shown in Table 6.

As shown in Table 7, apart from winter, pathogenic *V*. *parahaemolyticus* were detected in samples obtained from both aquafarms and retail markets in all seasons. The positive rate of pathogenic *V*. *parahaemolyticus* in aquafarms was greater than that in samples obtained from retail markets in all seasons, being significantly different only during autumn (*P* < 0.05). The highest density of pathogenic *V*. *parahaemolyticus* (240 MPN/g) was observed in samples obtained from retail markets during spring.

## 4. Discussion

The Zhoushan archipelago is rich in seafood resources, including *Anadara subcrenata*, a type of bivalve seashell consumed raw or semi-cooked by from the residents in Zhoushan. Owing to its special physical structure and soil environment preference, *Anadara subcrenata* can easily enrich *V. parahaemolyticus* in seawater [5]. Therefore, the risk of food-borne illnesses caused by *V. parahaemolyticus* through *Anadara subcrenata* is relatively high [13]. With the rapid development of the Chinese economy, the consumption of seafood has increased greatly, not only in the coastal cities of China, but also in mainland China. *V. parahaemolyticus* distribution in *Anadara subcrenata* from the eastern coastal cities of China has been reported previously [14, 15]. Using information relating to *V. parahaemolyticus* exposure from COVISS and CDC estimates that 62% of all *V. parahaemolyticus* illness cases are caused by consumption of raw oysters. US-FDA-predicted mean levels of total and pathogenic V. *Parahaemolyticus* in raw oysters at-harvest were 5.0–2100.0 MPN/g and 1.3–15.0 MPN/g in summer, <52.0 MPN/g and <0.1 MPN/g in winter.

In this study, we analyzed 316 *Anadara subcrenata* samples obtained during all four seasons from both aqua farms and retail markets in the Zhoushan area. The prevalence status of total *V. parahaemolyticus* in *Anadara subcrenata* observed in this study (83.86%) was greater than those reported previously in studies of samples from mainland China. The level of contamination of the total *V. parahaemolyticus* ranged from 0.3 to 2400.0 MPN/g with the median 40.0 MPN/g, concurrent with those reported previously in many other coastal cities in China [6, 16, 17].

The present results indicate that the positive rate of total *V. parahaemolyticus* in all four seasons indicated a high level of contamination (83.86%), comparing the results of Han [10] and Suffredini et al. [13], thereby reflecting a relatively high level of total *V. parahaemolyticus* contamination of the sea area of Zhoushan. This result shows an obvious seasonal distribution similar to those reported previously [3, 10, 18]. The level of contamination increased from spring and peaked in summer, reduced in autumn, and was the lowest in winter. This temporal distribution of total *V. parahaemolyticus* reflected the effect of water temperature [19]. Some researchers found that in winter, *V. Parahaemolyticus* were not detected in seawater samples, but some were detected in seawater sediments. It could be speculated that *V. Parahaemolyticus*' possible survival mechanism was: *V. Parahaemolyticus*' survives in seawater sediments in winter, proliferates after the temperature rises in summer, and then pollutes seawater and seafood.

In contrast with many studies [20, 21] reporting that the transportation and storage of seashells may increase contamination by *V. parahaemolyticus*; the present data indicate that aquafarms have a significantly higher abundance and prevalence (*P* < 0.01) of *V. parahaemolyticus* than retail markets. These findings indicate that aquafarms are the primary source of *V. parahaemolyticus* contamination in Zhoushan, and the spread of *V. parahaemolyticus* was effectively controlled during transport and storage of *Anadara subcrenata*. The *Anadara subcrenata* acquired from aquafarm was transported in 4°C to the main retail markets in Zhoushan. The authorities shall effectively monitor the pollution of seafood and its by-products, ban fishing or close farms in highly polluted sea areas, and prevent high-risk foods from entering the retail market. *V. Parahaemolyticus* is sensitive to low temperatures, so iced seafood is considered to have little chance of transmitting *V. parahaemolyticus*. Because *V. Parahaemolyticus* is not heat resistant, it is the most effective way to prevent *V. Parahaemolyticus* poisoning when food is well cooked.

In certain cases, the prevalence of pathogenic *V. parahaemolyticus* tended to increase in samples contaminated with relatively high levels of total *V. parahaemolyticus* [22]. The positive rate of pathogenic *V. parahaemolyticus* in this study was 9.43%, which was relatively higher than that in numerous cities in China [18]. Pathogenic *V. parahaemolyticus* was detected in all the seasons except in winter. However, winter may not be considered an absolutely safe season [12]. The highest levels of *tdh*- and *trh*-positive *V. parahaemolyticus* were observed during spring, which may be associated with the uneven contribution of sample numbers [4]. Samples collected from retail markets presented higher levels of pathogenic *V. parahaemolyticus* contamination. This result may be associated with a reduction in total *V. parahaemolyticus* density or with differences in the survival and tolerance of virulent and nonvirulent strains during transportation and storage.

Antimicrobials are overused in the treatment of infectious diseases in the aquaculture industry, which leads to the extensive use of antimicrobials that has led to the development of antimicrobial resistance among pathogens in aquatic products and has rendered antimicrobials ineffective. *V. parahaemolyticus* has been reported to have resistance to ampicillin, amikacin, kanamycin, tetracycline, ceftazidime, and cefotaxime [4, 20].

## 5. Conclusions

Our study's results highlight both total and pathogenic *V*. *parahaemolyticus* in two food supply chains of Zhoushan across all four seasons, suggesting the need to improve strategies to prevent the occurrence of diseases [4, 23] transmitted via consumption of *Anadara subcrenata* contaminated with pathogenic *V. parahaemolyticus*. The exceeding of restrictions set by Chinese Food Safety Standards GB29221 on ready-to-eat seafood, put Chinese consumers at a high risk with *V*. *Parahaemolyticus*-related gastroenteritis. Although most environmental *V*. *Parahaemolyticus* separations are nonpathogenic, consumers should still pay attention to and ensure that oysters are properly cooked before consumption. In addition, in order to prevent cross-contamination of wet markets and supermarkets, important measures must be taken, including the good hygiene practices in the handling of goods and the cleanliness of handlers and display areas. Furthermore, the continuous monitoring of the *V*. *Parahaemolyticus* prevalence and antibiotics should be carried on which it serves to derive data for risk assessment and to control the risk of food-borne diseases caused by *V. parahaemolyticus* in Zhoushan.

## Figures and Tables

**Table 1:** Number of samples collected during June 2013 to March 2015.

		Market categories	
		Retail market	Aquafarm	Total
Seasons	Spring	37	38	75
	Summer	39	60	99
	Autumn	38	40	78
	Winter	34	30	64
Total		148	168	316

**Table 2:** Primers and probes for multiplexed polymerase chain reaction-based detection of total and pathogenic *Vibrio parahaemolyticus* (Ward and Bej [12]).

Target gene	Primer or probe	Sequence^a^	Amplicon size (bp)
*tlh*	F-*tlh*	5′-AAA GCG GAT TAT GCA GAA GCA CTG-3′	450
	R-*tlh*	5′-GCT ACT TTC TAG CAT TTT CTC TGC-3′	
	P-tl952	5′-TEXR-AAG AAC TTC ATG TTG ATG ACA CT-BHQ2-3′^b^	
*tdh*	F-*tdh*170DG	5′-GTA RAG GTC TCT GAC TTT TGG AC-3′	229
	R-*tdh*403	5′-CTA CAG AAT YAT AGG AAT GTT GAA G-3′	
	P-*tdh*-341R	5′-CY5-ATT TTA CGA ACA CAG CAG AAT GA-Iowa Black-RQ-3′^c^	
*trh*	F-*trh*82	5′-CCA TCM ATA CCT TTT CCT TCT CC-3′	207
	R-*trh*287	5′-ACY GTC ATA TAG GCG CTT AAC-3′	
	P-*trh*275	5′-TET-TAT TTG TYG TTA GAA ATA CAA CAA T-BHQ1-3′^d^	

^a^Y = C or T; M = A or C; R = G or A. ^b^TexR, sulforhodamine 101 (Texas Red) fluorescent dye; BHQ2, Black Hole-2 quencher dye. ^c^Cy5, carbocyanine fluorescent dye; Iowa Black-RQ, Iowa Black quencher dye. ^d^TET, tetrachloro-6-carboxyfluorescein fluorescent dye.

**Table 3:** Contamination levels of total *Vibrio parahaemolyticus* in *Anadara subcrenata* in different seasons.

Seasons	Number of samples	No. of positive samples	Positive rate (%)	Percentage of samples with total *V. parahaemolyticus* (MPN/g)					Densities of *tlh* positive samples (MPN/g)	
				None detected (%)	<10 (%)	10–10^2^ (%)	10^2^–10^3^ (%)	>10^3^ (%)	Median	Min–max
Spring	75	56	74.67	25.33	28.00	34.67	4.00	8.00	20.00	0.36–2400
Summer	99	98	98.99	1.01	1.01	35.35	28.28	34.34	210.00	7.40–2400
Autumn	78	69	88.46	11.54	10.26	44.87	14.10	19.23	43.00	0.61–2400
Winter	64	42	65.63	34.38	64.06	1.56	0.00	0.00	0.92	0.30–15
Total	316	265	83.86^*∗*^	16.14	22.47	30.70	13.29	17.41	42.00^#^	0.30–2400

^*∗*^The positive rate of four seasons was in significant difference (*χ*^2^ = 38.371, df = 3, *P* < 0.01). ^#^The contamination level of total *V. parahaemolyticus* of four seasons was in significant difference (*χ*^2^ = 122.797, df = 3, *P* < 0.001).

**Table 4:** Prevalence of contamination of total *Vibrio parahaemolyticus* in *Anadara subcrenata* in different markets.

Markets	Number of samples	No. of positive samples	Positive rate (%)	Percentage of samples with total V. parahaemolyticus (MPN/g)					Densities of *tlh* positive samples (MPN/g)	
				None detected (%)	<10 (%)	10–10^2^ (%)	10^2^–10^3^ (%)	>10^3^ (%)	Median	Min–max
Aqua farm	168	156	92.85	7.14	21.43	30.95	17.26	23.21	53.00	0.36–2400
Retail market	148	109	73.64	26.35	23.65	30.41	8.78	10.81	24.00	0.30–2400
Total	316	265	83.86^*∗*^	16.14	22.47	30.70	13.29	17.41	42.00^#^	0.30–2400

^*∗*^The positive rate of two markets was in significant difference (*P* < 0.01). ^#^The contamination level of total *V. parahaemolyticus* of four seasons was in significant difference (*P* < 0.01).

**Table 5:** Prevalence of contamination of pathogenic *Vibrio parahaemolyticus* in *Anadara subcrenata* in different seasons.

Seasons	Number of samples	No. of positive samples	Positive rate (%)	Number of samples with pathogenic *V. parahaemolyticus* (MPN/g)					Densities of *tdh* or *tlh* positive samples (MPN/g)	
				None detected	<10	10–10^2^	10^2^–10^3^	>10^3^	Median	Min–max
Spring	56	10	17.85^*∗*^	46	7	2	1	0	3.3	0.92–240
Summer	98	8	8.16^*∗*^	90	5	2	1	0	3.6	0.36–210
Autumn	69	7	10.14^*∗*^	58	5	2	0	0	9.2	3.00–23.00
Winter	64	0	0	64	0	0	0	0	—	—
Total	265	25	9.43	240	17	6	2	0	6.4	0.30–240

^*∗*^The positive rate of pathogenic *V. parahaemolyticus* from different seasons was significantly different (*P* < 0.05).

**Table 6:** Prevalence of contamination of pathogenic *Vibrio parahaemolyticus* in *Anadara subcrenata* in different markets.

Markets	Number of samples	No. of positive samples	Positive rate (%)	Number of samples with pathogenic *V. parahaemolyticus* (MPN/g)					Densities of *tdh* or *tlh* positive samples (MPN/g)	
				None detected	<10	10–10^2^	10^2^–10^3^	>10^3^	Median	Min–max
Aqua farm	109	9	8.26^*∗*^	100	6	2	1	0	27	0.30–240.00
Retail market	156	16	10.26^*∗*^	140	11	4	1	0	53	0.36–240.00
Total	265	25	9.43	240	17	6	2	0	6.4	0.30–240

^*∗*^The positive rate of pathogenic *V. parahaemolyticus* between different markets had no significant difference (*P* > 0.05).

**Table 7:** Prevalence of contamination of pathogenic *Vibrio parahaemolyticus* in *Anadara subcrenata* among different seasons and markets.

Seasons	Aqua farm			Retail market			*χ* ^2^ value	*P* value
	Number of samples	Positive rate %	Median of *tdh* or *tlh* positive samples (MPN/g)	Number of samples	Positive rate %	Median of *tdh* or *tlh* positive samples (MPN/g)		
Spring	5	23.81	21	5	15.15	1.1	2.667	0.072
Summer	5	13.16	30	3	5.00	3	2.457	0.087
Autumn	6	19.35	9.25	1	2.63	7.4	4.726	<0.05^*∗*^
Winter	0	0	0	0	0	0	—	—
*χ* ^2^ value		2.115			3.021			
*P* value		0.099			<0.05			

^*∗*^The positive rate of pathogenic *V. parahaemolyticus* between different markets was significantly different only in autumn (*P* < 0.05).

## Data Availability

The data used to support the findings of this study are available from the corresponding author upon request.

## Additional Points

(1) The MPN-PCR method was used to estimate total and pathogenic *V. parahaemolyticus*. (2) High positive rate (83.86%) of total VP was detected in *Anadara subcrenata* in Zhoushan area. (3) All samples were classified on the basis of four seasons, two sampling methods, and five levels of MPN values. (4) Opposite results of total VP and pathogenic VP were obtained from different sampling methods.

## References

[1] Chakraborty R. D., Surendran P. K., Joseph T. C. (2008). Isolation and characterization of *Vibrio parahaemolyticus* from seafoods along the southwest coast of India. *World Journal of Microbiology and Biotechnology*.

[2] Hara-Kudo Y., Sugiyama K., Nishibuchi M. (2003). Prevalence of pandemic thermostable direct hemolysin-producing *Vibrio parahaemolyticus* O3 : K6 in seafood and the coastal environment in Japan. *Applied and Environmental Microbiology*.

[3] Miwa N., Kashiwagi M., Kawamori F. (2006). Levels of *Vibrio parahaemolyticus* and thermostable direct hemolysin gene-positive organisms in retail seafood determined by the most probable number-polymerase chain reaction (MPN-PCR) method. *Journal of the Food Hygienic Society of Japan (Shokuhin Eiseigaku Zasshi)*.

[4] Parveen S., Hettiarachchi K. A., Bowers J. C. (2008). Seasonal distribution of total and pathogenic *Vibrio parahaemolyticus* in Chesapeake Bay oysters and waters. *International Journal of Food Microbiology*.

[5] Liu X. M., Chen Y., Guo Y. C., Wang Z. T. (2008). Foodborne diseases outbreaks in 2005-report of national foodborne diseases surveillance network in China. *Chinese Journal of Food Hygiene*.

[6] Chen Y., Guo Y., Wang Z. (2010). Foodborne disease outbreaks in 2006 report of the national foodborne disease surveillance network, China. *Journal of Hygiene Research*.

[7] Lee L.-H., Ab Mutalib N.-S., Law J. W.-F., Wong S. H., Letchumanan V. (2018). Discovery on antibiotic resistance patterns of *Vibrio parahaemolyticus* in Selangor reveals carbapenemase producing *Vibrio parahaemolyticus* in marine and freshwater fish. *Frontiers in Microbiology*.

[8] Nordstrom J. L., Vickery M. C. L., Blackstone G. M., Murray S. L., DePaola A. (2007). Development of a multiplex real-time PCR assay with an internal amplification control for the detection of total and pathogenic *Vibrio parahaemolyticus* bacteria in oysters. *Applied and Environmental Microbiology*.

[9] Muntada-Garriga J. M., Rodriguez-Jerez J. J., Lopez-Sabater E. I., Mora-Ventura M. T. (1995). Effect of chill and freezing temperatures on survival of *Vibrio parahaemolyticus* inoculated in homogenates of oyster meat. *Letters in Applied Microbiology*.

[10] Han H., Li F., Yan W. (2015). Temporal and spatial variation in the abundance of total and pathogenic *Vibrio parahaemolyticus* in shellfish in China. *PLoS One*.

[11] Ma C., Deng X., Ke C. (2014). Epidemiology and etiology characteristics of foodborne outbreaks caused by *Vibrio parahaemolyticus* during 2008–2010 in Guangdong province, China. *Foodborne Pathogens and Disease*.

[12] Ward L. N., Bej A. K. (2006). Detection of *Vibrio parahaemolyticus* in shellfish by use of multiplexed real-time PCR with TaqMan fluorescent probes. *Applied and Environmental Microbiology*.

[13] Suffredini E., Mioni R., Mazzette R. (2014). Detection and quantification of *Vibrio parahaemolyticus* in shellfish from Italian production areas. *International Journal of Food Microbiology*.

[14] Wu Y., Wen J., Ma Y., Ma X., Chen Y. (2014). Epidemiology of foodborne disease outbreaks caused by *Vibrio parahaemolyticus*, China, 2003–2008. *Food Control*.

[15] Xu X., Cheng J., Wu Q., Zhang J., Xie T. (2016). Prevalence, characterization, and antibiotic susceptibility of *Vibrio parahaemolyticus* isolated from retail aquatic products in North China. *BMC Microbiology*.

[16] Letchumanan V., Pusparajah P., Tan L. T.-H., Yin W.-F., Lee L.-H., Chan K.-G. (2015). Occurrence and antibiotic resistance of *Vibrio parahaemolyticus* from shellfish in Selangor, Malaysia. *Frontiers in Microbiology*.

[17] Wagley S., Koofhethile K., Wing J. B., Rangdale R. (2008). Comparison of *V. parahaemolyticus* isolated from sea foods and cases of gastrointestinal disease in the UK. *International Journal of Environmental Health Research*.

[18] Saito S., Iwade Y., Tokuoka E. (2015). Epidemiological evidence of lesser role of thermostable direct hemolysin (TDH)-Related hemolysin (TRH) than TDH on *Vibrio parahaemolyticus* pathogenicity. *Foodborne Pathogens and Disease*.

[19] DePaola A., Nordstrom J. L., Bowers J. C., Wells J. G., Cook D. W. (2003). Seasonal abundance of total and pathogenic *Vibrio parahaemolyticus* in Alabama oysters. *Applied and Environmental Microbiology*.

[20] Letchumanan V., Chan K. G., Lee L. H. (2014). *Vibrio parahaemolyticus*: a review on the pathogenesis, prevalence and advance molecular identification techniques. *Frontiers in Microbiology*.

[21] Smith C., Painter J., Tauxe R. (2004). *Oyster Consumption Accounts for a Majority of Vibrio Parahaemolyticus Infections in the United States*.

[22] Fernandez-Piquer J., Bowman J. P., Ross T., Tamplin M. L. (2011). Predictive models for the effect of storage temperature on *Vibrio parahaemolyticus* viability and counts of total viable bacteria in Pacific oysters (*Crassostrea gigas*). *Applied and Environmental Microbiology*.

[23] Raghunath P. (2015). Roles of thermostable direct hemolysin (TDH) and TDH-related hemolysin (TRH) in *Vibrio parahaemolyticus*. *Frontiers in Microbiology*.

